# Role of fantasy in emotional clarity and emotional regulation in empathy: A preliminary study

**DOI:** 10.3389/fpsyg.2022.912165

**Published:** 2022-11-17

**Authors:** Shoichi Shiota, Michio Nomura

**Affiliations:** ^1^Faculty of Human Relations, Tokai Gakuin University, Kakamigahara City, Gifu, Japan; ^2^Graduate School of Education, Kyoto University, Kyoto, Japan

**Keywords:** empathy, metacognition, fantasy, emotional clarity, emotional regulation

## Abstract

Fantasy is the experience of identifying with characters in movies, novels, plays, and other fictional situations. In social contexts, individuals take on the perspective of others by sensing their emotions through empathy. During this process, perspective-taking and emotional sharing affect one’s metacognition, which deals with the distinction between and the understanding of one’s emotions (clarity) and their regulation (repair); previous studies have primarily focused on these processes. However, perspective-taking—considering another individual’s viewpoint—requires one to imagine their outlook; it also induces emotional responses. This study examined the role of fantasy in clarity and repair in metacognition, for which data derived from 475 Japanese participants were analyzed. The results of the Interpersonal Reactivity Index showed that fantasy was positively associated with clarity and repair in the Trait Meta-Mood Scale; these relationships were moderated by perspective-taking and personal distress. Our results revealed that the emotions experienced within oneself might be understood as the distinction between “imagining” (through their imagination; e.g., internal or mental pictures) and “imaging” (from an image; e.g., external pictures). Individuals imagine their immersion into others using lower-level automatic body sensations (emotional contagion), and the accompanying negative emotions are regulated by metacognition.

## Introduction

Metacognition is defined as the cognitive function of objectively monitoring one’s inner experiences and emotional events, which vary momentarily (Kabat-Zinn, 1994). It consists of three aspects: clarity, which is related to the distinction between and understanding of one’s emotions; repair, which is related to emotional regulation and improving negative emotions; and attention, which is related to defining one’s feelings (Gohm and Clore, 2002). Metacognition refers to the activities involved in thinking and forming integrated ideas about oneself and others (Bonfils et al., 2019). Empathy is defined as the ability to experience and understand the momentary emotional state of another individual (Salazar-López et al., 2015), and each component in empathy is closely contributed to metacognition. Fantasy is one of the empathy components that can be defined as the mental experiences associated with a tendency to identify with characters in movies, novels, plays, and other fictional situations (Davis, 1980, 1983). It is considered a central component of meaning (Weibel et al., 2018). According to Weibel et al. (2018), “Fantasy offers a way of using cognitive functions for mental exploration and metacognition of counterfactual or expanded realms beyond the perceived ‘real world.” Therefore, it is suggested that fantasy in empathy is contributed to metacognition. However, previous studies have only examined the effects of perspective-taking and emotional sharing, which are components of empathy, on metacognition regarding clarity and repair (e.g., Decety and Meyer, 2008). Accordingly, perspective-taking—understanding the viewpoint of others—requires one to imagine those perspectives. Hence, emotions are experienced within oneself by imagining those of others (Eisenberg et al., 1988; Barrett, 1992; Stotland and Smith, 1994; McInnis, 2014). Based on these evidences, it is not only the relationships among fantasy, perspective-taking, and metacognition, that are suggested but also those of fantasy, emotional sharing, and metacognition. However, the details of these processes and the role of fantasy in them have not been studied extensively.

Salovey et al. (1995) developed an “individual difference” based measure of metacognition called the Trait Meta-Mood Scale (TMMS). The scale consists of three aspects: clarity, repair, and attention (Gohm and Clore, 2002). A previous study demonstrated a significant positive correlation between the inter-individual differences in scores for fantasy and clarity (Gohm and Clore, 2002). Thompson et al. (2017) reported that individuals with major depressive disorder and social anxiety disorder, including those with abnormal fantasies (Rude et al., 2002; Moscovitch et al., 2018), scored lower than healthy subjects for both clarity and repair in the TMMS; both functions were also negatively correlated with clinical symptoms. Likewise, another study found a significant positive correlation between the inter-individual differences in the scores for fantasy and repair (Shi and Wang, 2007), while Eisenberg et al. (1988) reported a negative correlation between the inter-individual differences in scores for fantasy and sadness. These results suggest that fantasy directly contributes to clarity and repair in metacognition. However, the mechanisms of these processes are unknown.

Empathy is an important function of recognizing the emotional and mental states of others. The trait empathy of individuals is measured using the Interpersonal Reactivity Index (IRI; Davis, 1980; Himichi et al., 2017). The IRI is a self-report measure of the following aspects of empathy: fantasy and perspective-taking (cognitive empathy); and personal distress and empathic concern (affective empathy; Davis, 1983). According to Davis (1980, 1983), perspective-taking items reflect the respondent’s ability to adopt the perspectives of others. It leads one to anticipate the helping behaviors and reactions of others. Personal distress indicates that the respondent experiences discomfort and anxiety upon witnessing others having negative experiences. Empathic concern assesses a respondent’s tendency to experience feelings of warmth, compassion, and concern for others undergoing negative experiences. By focusing on fantasy’s relationship to perspective-taking and personal distress, Davis (1980) found that fantasy contributes to both perspective-taking and personal distress. McInnis (2014) revealed that high fantasy-orientated children had better scores for cognitive empathy and affective empathy than low fantasy-orientated children. In recent years, numerous studies have examined the relationships between perspective-taking and clarity and personal distress and repair (e.g., Decety and Lamm, 2011). Fernández-Abascal and Martín-Díaz (2019) reported a positive correlation between perspective-taking in the IRI and clarity in the TMMS. Eckland et al. (2018) observed a significant positive correlation between cognitive empathy—including fantasy and perspective-taking—and clarity when considering the emotions generated by the observer, thereby suggesting that clarity in metacognition is affected by perspective-taking. A recent study that examined the relationship between personal distress and repair found a negative correlation between personal distress in the IRI and repair in the TMMS (Fernández-Abascal and Martín-Díaz, 2019). Repair in metacognition is affected by personal distress. In summary, these findings indicate that fantasy in empathy is positively associated with clarity and repair in metacognition; furthermore, this relationship is moderated by perspective-taking and personal distress.

Based on the literature, the relationships among fantasy in empathy, perspective taking in empathy, and clarity in metacognition are suggested. The relationships between fantasy in empathy, emotional sharing in empathy, and repair in metacognition are also suggested. Nevertheless, few studies have examined the details of these processes and the role of fantasy in them. The present study has academic value as it clarifies the role of fantasy in emotional clarity and emotional regulation while practicing empathy. We hypothesized the following:

*H1*: Based on previous studies (McInnis, 2014; Thompson et al., 2017; Fernández-Abascal and Martín-Díaz, 2019), fantasy is positively associated with clarity in the Japanese version of the TMMS (TMMS-J), and this relationship is moderated by perspective-taking.

*H2*: Based on previous studies (Shi and Wang, 2007; McInnis, 2014; Fernández-Abascal and Martín-Díaz, 2019), fantasy is positively associated with repair in the TMMS-J, and the relationship is moderated by personal distress.

## Materials and methods

### Participants

We used CrowdWorks^1^ to recruit 481 healthy Japanese participants who spoke Japanese as their native language and had no history of psychiatric disorders. They completed an online questionnaire distributed by Qualtrics, an online research company.^2^ First, the participants answered questions about demographics such as age, sex, and presence or absence of history of psychiatric disorders. Second, they answered the self-report questionnaires. Six participants were excluded from the analysis for providing incorrect responses. Thus, the final data of 475 healthy Japanese participants (242 women; aged 20–75, mean age 39.4 years, standard deviation [*SD*] = 10.50) were available for analysis. We calculated the sample size by conducting a power analysis using G*Power (Faul et al., 2007) with the correlation coefficient (0.20), which was used to establish the criteria-related validity between the TMMS and other scales in a previous study (Fernández-Berrocal et al., 2004). Power analysis results indicated that at least 436 participants would be necessary for our analysis (effect size = *r*, α = 0.01, Power = 0.95); thus, our sample size was sufficient. Before completing the questionnaire, all the participants provided their written informed consent.

### Self-report questionnaires

#### Japanese version of the trait meta-mood scale

The original TMMS was developed by Salovey et al. (1995) to examine the ability of individuals to understand their moods and feelings, monitor the degree to which people moderate their moods, and determine the relationship between feelings and thoughts. The TMMS consists of three subscales: “attention,” “clarity,” and “repair.” In this context, “attention” is an individual’s ability to define their feelings (e.g., “I pay a lot of attention to how I feel”); “clarity” is the ability to identify differences in emotions (e.g., “I am usually very clear about my feelings”); and “repair” is the capacity to improve negative emotions when required (e.g., “I try to think good thoughts, no matter how bad I feel”). The TMMS-J was translated and back-translated by native English speakers. We selected the following items from each subscale of the original TMMS based on pilot studies: attention: 7, 8, 13, 15, 35, 38, 41, and 46; clarity: 9, 12, 24, 28, 33, 37, 42, and 48; and repair: 2, 3, 10, 16, 17, 30, and 43. We conducted a confirmatory factor analysis of the TMMS-J scores. The result indicated a moderate to good fit to the model with the three-factor structure (χ^2^ (227) = 942.52, *p* < 0.001, GFI = 0.835, CFI = 0.814, RMSEA = 0.082). The Cronbach’s alpha was α = 0.81 for attention, α = 0.86 for clarity, and α = 0.71 for repair, thus indicating adequate internal consistency for each TMMS-J subscale in this study. The TMMS-J also had good internal consistency, equal to that of previous studies (attention; *α* = 0.76–0.86; clarity *α* = 0.73–0.87; repair α = 0.60–0.82; Salovey et al., 1995; Fernández-Berrocal et al., 2004; Bugay et al., 2014). The items were measured on a 5-point Likert scale ranging from 1 (“Does not describe me well”) to 5 (“Describes me very well”).

#### Japanese version of the interpersonal reactivity index

The Japanese version of IRI (IRI-J; Davis, 1980; Himichi et al., 2017) is a 28-item questionnaire widely used for the multidimensional assessment of trait empathy. It consists of four subscales: perspective taking, personal distress, empathic concern, and fantasy. Each of the four subscales comprises seven items that are assessed on a 5-point Likert scale ranging from 1 (“Does not describe me well”) to 5 (“Describes me very well”). Each subscale has a minimum total score of 7 or a maximum total score of 35. Perspective-taking evaluates an individual’s tendency to adopt the psychological viewpoints of others (e.g., “I sometimes try to understand my friends better by imagining how things look from their perspective”). Personal distress is self-oriented and associated with aversive emotional responses in the observer (e.g., “I tend to lose control during emergencies”). Conversely, the empathic concern is other-oriented and relates to feelings of compassion and sympathy for the observed individual (e.g., “When I see someone being taken advantage of, I feel protective toward them”). Fantasy entails the participants’ abilities to transpose (immerse) themselves in fictional situations (e.g., “When I am reading an interesting story or novel, I imagine how I would feel if the events in the story were happening to me”). The mean and SDs for TMMS-J and IRI-J are shown in Table 1. These values are similar to those of Himichi et al. (2017).

**Table 1:** Means and *SD*s for each scale (*n* = 475).

		Mean	*SD*
	Age	39.39	10.50
TMMS-J	Repair	23.04	4.60
	Attention	28.61	4.34
	Clarity	23.96	6.11
IRI-J	PT	21.58	4.35
	PD	23.12	5.48
	EC	24.40	4.82
	FS	22.63	4.68

*SD*, standard deviation; TMMS-J, The Japanese version of the Trait Meta Mood Scale; IRI-J, The Japanese version of the Interpersonal Reactivity Index.

## Results

### Hierarchical multiple regression analyses

Hierarchical multiple regression analyses were performed to examine the main effect of each subcomponent of empathy and metacognition, along with their interactive effects. Gender and age were entered into the model as the control variables in Step 1. The main effect and interaction of each subscale in the IRI-J were entered in Step 2. The results are summarized in Table 2. After controlling for the demographic variables, the main effect and interaction between each subscale in the IRI-J were explored as potential predictors of the variance in clarity in the TMMS-J (Δ*R*^2^ = 0.34; *F* [10, 462] = 22.64; *p* < 0.05). The main effect of personal distress in the IRI-J (*β* = −0.58; *p* < 0.05; 95% CI = [−0.729, −0.554]) and interaction between perspective-taking and fantasy in the IRI-J (*β* = 0.11; *p* < 0.05; 95% CI = [0.006, 0.055]) significantly predicted clarity in the TMMS-J. This interaction was examined using a simple slope analysis.

**Table 2:** Hierarchical multiple linear regression predicting clarity and repair in the TMMS-J from each subscale in the IRI-J (*N* = 475).

	Clarity in the TMMS-J							
	Step 1				Step 2			
	*B*	*β*	*t*-Value	*p*-Value	*B*	*β*	*t*-Value	*p*-Value
Intercept	18.23		11.77	*p* < 0.05	29.10		12.74	*p* < 0.05
Sex	0.94	0.08	1.65	*p* = 0.10	2.11	0.17	4.35	*p* < 0.05
Age	0.11	0.19	4.04	*p* < 0.05	0.60	0.10	2.55	*p* < 0.05
Fantasy in the IRI-J					0.02	0.07	0.45	*p* = 0.13
Perspective-taking in the IRI-J					0.70	0.50	1.15	*p* = 0.25
Personal distress in the IRI-J					−0.64	−0.60	−14.38	*p* < 0.05
Empathic concern in the IRI-J					0.09	0.07	1.58	*p* = 0.12
Fantasy × Perspective-taking					0.03	0.11	2.49	*p* < 0.05
Fantasy × Personal distress					0.01	0.05	1.31	*p* = 0.19
Fantasy × Empathic concern					0.04	0.02	0.36	*p* = 0.72
Perspective-taking × Personal distress					<0.01	≤0.01	−0.21	*p* = 0.83
Perspective-taking × Empathic concern					−0.02	−0.07	−1.58	*p* = 0.12
Personal distress × Empathic concern					−0.02	−0.07	−1.63	*p* = 0.10
Δ*R*^2^	0.03*				0.34			
*R* ^2^	0.03*				0.37			
	**Repair in the TMMS-J**							
	**Step 1**				**Step 2**			
	***B***	***β***	***t*-Value**	***p*-Value**	***B***	***β***	***t*-Value**	***p*-Value**
	21.00		17.74	*p* < 0.05	18.92		10.13	*p* < 0.05
	0.52	0.06	1.19	*p* = 0.23	0.79		2.01	*p* = 0.47
	0.03	0.08	1.59	*p* = 0.11	0.22	0.21	4.55	*p* < 0.05
					0.07	0.08	1.7	*p* = 0.09
					0.22	0.21	4.55	*p* < 0.05
					−0.30	−0.35	−8.15	*p* < 0.05
					0.11	0.12	2.55	*p* < 0.05
					<0.01	0.02	0.41	*p* = 0.68
					0.02	0.11	2.32	*p* < 0.05
					−0.01	−0.04	−0.80	*p* = 0.43
					< 0.01	−0.01	−0.32	*p* = 0.75
					< 0.01	−0.01	−0.29	*p* = 0.77
					−0.01	−0.07	−1.55	*p* = 0.12
	0.01				0.26			
	0.01				0.25			

IRI-J, Japanese version of the Interpersonal Reactivity Index; TMMS-J, Japanese version of the Trait Meta Mood Scale.

**Figure 1:**
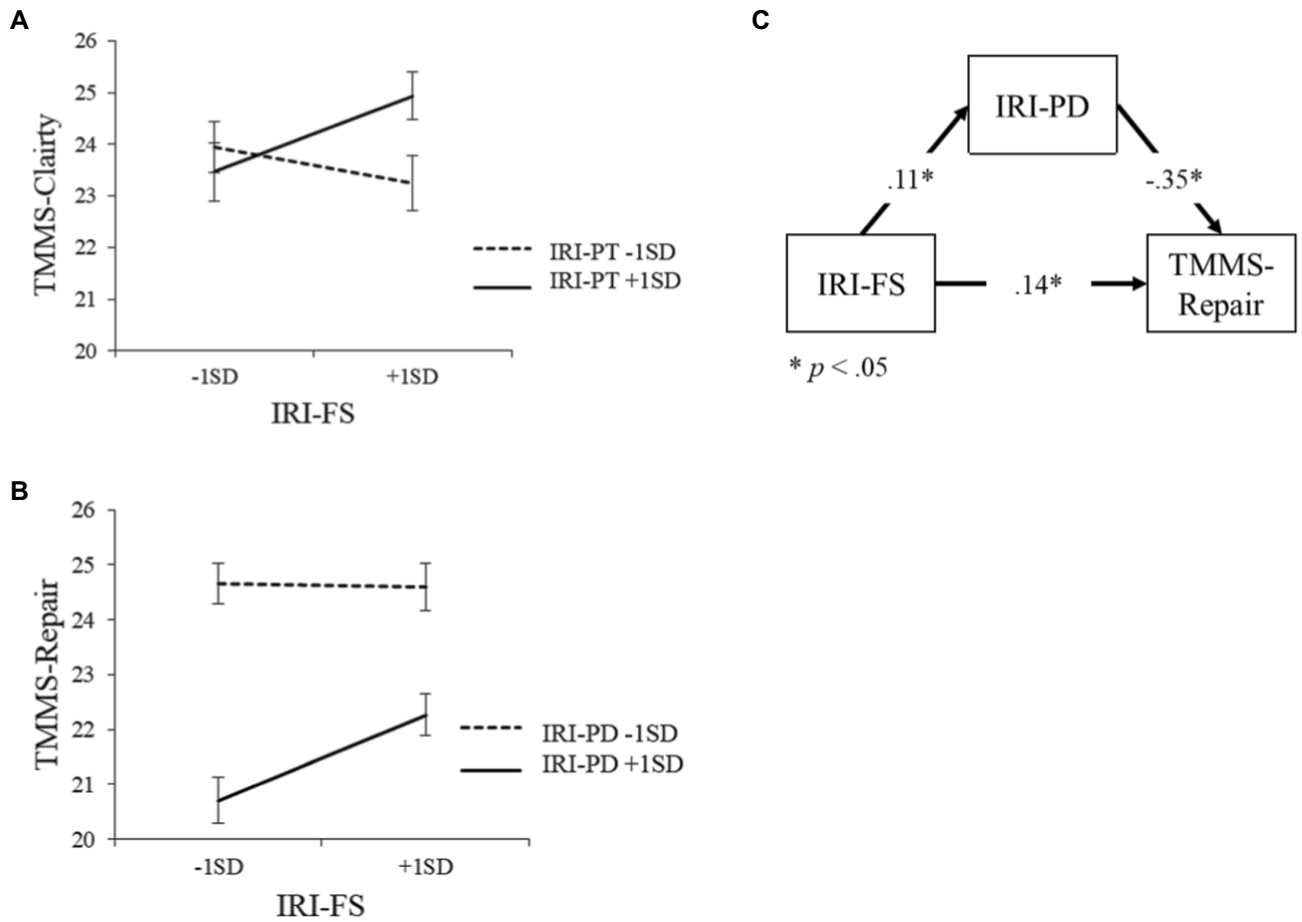
The relationship between fantasy, perspective-taking, personal distress, metacognitive clarity, and metacognitive repair. **(A)** Moderating effect of perspective-taking on the relationship between fantasy in the IRI-J and clarity in the TMMS-J. **(B)** Moderating effect of personal distress on the relationship between fantasy in the IRI-J and repair in the TMMS-J. **(C)** Mediation model of fantasy in the IRI-J, personal distress in the IRI-J, and repair in the TMMS-J.

Subsequently, based on a previous study (Takano et al., 2018), we split the moderator using “median ± 1SD” in the simple slope analysis. The results showed that fantasy was positively associated with clarity in the high-scores group for perspective-taking scores in the IRI-J (*β* = 0.12, *p* < 0.05). However, this relationship was not significant in the corresponding low-scores group (perspective-taking in the IRI-J; *β* = 0.06, *p* = 0.32; Figure 1A). After controlling for the demographic variables, the main effect and interaction of each subscale in the IRI-J were explored as significant predictors of the variance in repair (Δ*R*^2^ = 0.25; *F* [10, 462] = 22.64; *p* < 0.05). The main effect of perspective-taking (*β* = 0.21; *p* < 0.05; 95% CI = [0.127, 0.321]), personal distress (*β* = −0.35; *p* < 0.05; 95% CI = [−0.369, −0.226]), empathic concern (*β* = 0.12; *p* < 0.05; 95% CI = [0.026, 0.202]), and the interaction between personal distress and fantasy (*β* = 0.11; *p* < 0.05; 95% CI = [0.003, 0.032]) significantly predicted repair.

This significant interaction was further examined *via* a simple slope analysis. The results showed that fantasy was positively associated with repair in the low-scores group for personal distress (*β* = 0.17; *p* < 0.05). However, this relationship was not significant in the corresponding high-scores group (personal distress in the IRI-J; *β* = −0.01, *p* = 0.91; Figure 1B). After controlling for the demographic variables, the main effect and interaction of each subscale in the IRI-J were explored as significant predictors of the variance in attention in the TMMS-J (Δ*R*^2^ = 0.23; *F*[10, 462] = 22.64; *p* < 0.05). The main effect of perspective-taking (*β* = 0.11; *p* < 0.05; 95% CI = [0.017, 0.203]), empathic concern (*β* = −0.31; *p* < 0.05; 95% CI = [−0.195, 0.364]), and fantasy (*β* = 0.20; *p* < 0.05; 95% CI = [0.104, 0.267]) significantly predicted attention.

### Mediation analysis

We next conducted a mediation analysis with fantasy as the independent variable, repair as the dependent variable, and personal distress as the mediating variance (Figure 1C). The results indicated that fantasy had a significant direct effect on repair (*β* = 0.14; *p* < 0.05). The relationship between fantasy and repair was significantly mediated by personal distress (Sobel-test, *Z* = 3.52; *p* < 0.05). Moreover, fantasy had a direct effect on personal distress (*β* = 0.11; *p* < 0.05), and personal distress had a direct effect on repair (*β* = −0.35; *p* < 0.05).

## Discussion

The results indicated that perspective-taking and personal distress contribute to clarity and repair, which is consistent with the findings of previous studies (e.g., Fernández-Abascal and Martín-Díaz, 2019). Notably, fantasy was positively associated with clarity when the perspective-taking score was high, thus confirming our first hypothesis (H1). Additionally, fantasy was positively associated with repair when the personal distress score was high, which confirmed our second hypothesis (H2). Further, the relationship between fantasy and repair was significantly mediated by personal distress. Notably, these results are not consistent with those of Decety and Lamm (2011). The pattern observed in our results suggests that comprehension of emotions is promoted by imagining the emotions of others.

According to Suzuki and Kino (2008), fantasy is a self-oriented function (e.g., the tendency to imagine being the other and thinking about “how I feel” as that person). Conversely, perspective-taking is an other-oriented function, and its purpose is to understand the observed individual (Suzuki and Kino, 2008). This cognitive process requires suppressing self-oriented thinking and considering the viewpoints of others (Suzuki and Kino, 2008). Previous studies (e.g., Decety and Meyer, 2008) focused on the effect of perspective-taking on clarity in metacognition. In this study, fantasy was positively associated with clarity when the perspective-taking score was high. A few studies (Taylor and Carlson, 1997; Eckland et al., 2018) provided similar findings. It is suggested that both fantasy and perspective-taking are essential components of distinguishing and understanding one’s emotions.

Gallese and Goldman (1998) hypothesized that affective empathy entails “simulation” processes. Humans mirror others’ emotional responses to observed and experienced emotions in affective empathy. Our results suggested double dissociation between these “simulation” processes. In the first process, the results indicated that fantasy directly contributes to repair in metacognition; previous studies support these findings. For example, Eisenberg et al. (1988) reported a negative correlation between fantasy and sadness, which is a reaction to a sympathy experience. Similarly, Ferguson and Olson (2013) reported that playing fantasy video games reduces stress and enhances children’s motivation, which is a function of resilience in the face of failure and the desire for social interaction. Previous and present findings established that fantasy reduces unpleasant emotions, enabling adaptive emotional regulation. In the second process, our results demonstrate that fantasy is related to emotional regulation, and this relationship is moderated by personal distress. Within the social cognitive domain, indicators of low self-other distinction are motor imitation and emotion contagion when we effectively take on the physical and emotional states of others (Eddy, 2022). Frequent emotion contagion may lead to emotional dysregulation; detachment from emotional experiences may help combat personal distress (Eddy, 2022). For example, during the last decade, there has been a growing body of research on the abnormality of fantasy and personal distress in empathy (e.g., Harari et al., 2010; New et al., 2012; De Meulemeester et al., 2021). Harari et al. (2010) held that higher-order cognitive empathy processes, including fantasy and perspective-taking, which are impaired in borderline personality disorder, fail to modulate lower-level automatic emotional contagion, thus leading to elevated emotional empathy. De Meulemeester et al.’s, 2021 study showed that the ability to distinguish one’s mental representations from those of others is a key feature of borderline personality disorder. Compared to healthy individuals, those with borderline personality disorder may immerse themselves in others more easily. This tendency may be due to the abnormality of affective empathy. Additionally, New et al. (2012) have shown that individuals with borderline personality disorder have abnormal experiences of personal distress. Personal distress has been described as affect sharing, similar to that seen in affective empathy but without a distinction between the self and other, thus resulting in self-oriented distress rather than an empathic reaction (Preston and De Waal, 2002; De Vignemont and Singer, 2006; Decety and Jackson, 2006). Based on previous studies (Gallese and Goldman, 1998; Harari et al., 2010; New et al., 2012; De Meulemeester et al., 2021; Sowden et al., 2022) and our results, an explanation for empathic personal distress may be that individuals imagine and immerse themselves in others’ experiences using lower-level automatic body sensations (emotional contagion), and the accompanying negative emotions are regulated by one’s metacognition. On the other hand, frequency of personal distress could contribute to whether or not one engages in successful empathic interaction. Therefore, it is necessary to examine the double dissociations between “simulation” processes in affective empathy and the roles of fantasy and personal distress.

Although the importance of fantasy has been suggested in earlier research on empathy (Stotland and Smith, 1994), recent studies that have examined the relationship between empathy and metacognition have mainly focused on the relationships between perspective-taking and clarity, and personal distress and repair (e.g., Decety and Lamm, 2011). However, the role of fantasy has not been considered thoroughly. Fantasy is like a canvas in one’s mind, on which one can draw characters such as themselves or others. This canvas immerses people in a comprehensive image of an event. It is important to consider that elevated fantasies may not always demonstrate a beneficial effect (e.g., distracting from emotions, which may not always be helpful).

This study had several limitations. First, the fantasy scale in this study was one of the subscales of the IRI. We could not use other questionnaires that measured various aspects of fantasy. Furthermore, our study did not use an experimental design, and the result was not derived while participants were practicing empathy. Therefore, in the future, it is necessary to conduct behavioral and fMRI-based experiments incorporating positive and negative valences. Second, healthy Japanese individuals were recruited to complete the online survey. Building on previous studies that have reported on the abnormality of metacognition and fantasy (e.g., Semerari et al., 2003; Rasmussen et al., 2017), future research must examine the role of fantasy in both healthy and mentally ill populations alike. Third, our results indicate that fantasy was not associated with repair in a group with high personal distress scores. It implies the strength of the negative effect that personal distress has on emotional regulation. For example, feeling high levels of others’ pain diminishes one’s ability to effectively carry out emotional self-regulation. Fourth, TMMS-J had only a moderately good model fit to the original three-factor structure. As previous review articles have suggested that there are cultural differences in the interpretation of and responses to emotions between people in Eastern and Western countries (De Vaus et al., 2018), it is of interest to conduct empirical research in different cultural contexts. Finally, metacognition is a comprehensive concept, with prior studies indicating the relationship between metacognition and autobiographical memory (e.g., Dimaggio et al., 2008). Hence, an experimental paradigm that includes the effects of autobiographical memory must be developed in future studies. Despite these limitations, this study offered valuable insights into the mechanisms of empathy.

## Data availability statement

The datasets presented in this study can be found in online repositories. The names of the repository/repositories and accession number(s) can be found in the article/Supplementary material.

## Ethics statement

The studies involving human participants were reviewed and approved by the ethics committee of the Graduate School of Education, at Kyoto University. Written informed consent to participate in this study was provided by the participants’ legal guardian/next of kin.

## Author contributions

SS, MN: conceived and designed the study and contributed to the writing of the manuscript. SS: performed the recruitment and survey. All authors contributed to the article and approved the submitted version.

## Funding

This study was supported by the Grant-in-Aid for the Japan Society for the Promotion of Science (JSPS) fellows (18 J01157).

## Conflict of interest

The authors declare that the research was conducted in the absence of any commercial or financial relationships that could be construed as a potential conflict of interest.

## Publisher’s note

All claims expressed in this article are solely those of the authors and do not necessarily represent those of their affiliated organizations, or those of the publisher, the editors and the reviewers. Any product that may be evaluated in this article, or claim that may be made by its manufacturer, is not guaranteed or endorsed by the publisher.

## Footnotes

^1^ https://crowdworks.co.jp/

^2^ https://www.qualtrics.com/jp/
