# Supplementary material for: Role of fantasy in emotional clarity and emotional regulation in empathy: A preliminary study
Source: Front Psychol. 2022 Nov 17;13:912165. doi: 10.3389/fpsyg.2022.912165 (PMC9714674; doi:10.3389/fpsyg.2022.912165)
Supplement: Supplementary file 1 [file Data_Sheet_1.PDF]

Supplementary Material

1 Supplementary Data

To examine the effect of fantasy on clarity and repair in metacognition in empathy. We developed a Japanese version of the TMMS (TMMS-J). The procedure and results are shown below.

Japanese Version of the Self-Consciousness Scale (SCS-J)

Based on a previous study (Salovey et al., 1995), we used the SCS-J. The SCS-J is based on the original SCS (Fenigstein et al., 1975; Sugawara, 1984) and is a 26-item questionnaire that assesses individual differences in private and public self-consciousness. Each subscale contains thirteen items scored on a 7-point Likert scale ranging from 1 (“Does not describe me well”) to 7 (“Describes me very well”). The term “private self-consciousness” refers to the tendency to think about and attend to more covert or hidden aspects of the self, such as thoughts or emotions of a personal nature that are not easily susceptible to public scrutiny (e.g., “I am often the subject of my own fantasies”). Conversely, public self-consciousness refers to the tendency to think about those aspects of the self that are on public display, such as qualities from which impressions are formed in the eyes of other people (e.g., “I am concerned about what other people think of me”). A previous study (Salovey et al., 1995) indicated that the private and public aspects of the SCS-J are positively correlated with attention in the TMMS-J.

Results

Development of the TMMS-J

We examined the criterion-related validity of the TMMS-J (Table 1). Attention in the TMMS-J was significantly correlated with public (r = .30, p < .05) and private (r = .59, p < .05) self-consciousness in the SCS-J. Additionally, repair in the TMMS-J was significantly correlated with private self-consciousness (r = .30, p < .05) in the SCS-J. We, therefore, concluded that these results replicate the criterion-related validity of Salovey et al.’s (1995) original TMMS.

Correlation Analyses

Correlation coefficients for each scale are shown in Table 2. We calculated the correlation coefficients between the IRI-J and the TMMS-J subscales to examine the relationship between inter-individual differences in empathy and metacognition. The results indicate that perspective taking in the IRI-J was significantly correlated with repair in the TMMS-J (r = .36; p < .05), attention in the TMMS-J (r = .28; p < .05), and clarity in the TMMS-J (r = .18; p < .05). Personal distress in the IRI-J was significantly correlated with repair in the TMMS-J (r = -.36; p < .05) and clarity in the TMMS-J (r = -.56; p < .05). Empathic concern in the IRI-J was significantly correlated with repair in the TMMS-J (r = .21; p < .05) and attention in the TMMS-J (r = .42; p < .05). Fantasy in the IRI-J was significantly correlated with repair in the TMMS-J (r = .14; p < .05) and attention in the TMMS-J (r = .31; p < .05). These results suggest that each of the subdomains of empathy directly affects attention and repair—which are

lower dimensions of metacognition—instead of clarity, which is a high order dimension of metacognition that often applies to a social context.
